# Are women more likely to engage in extra green behaviors in the workplace? Gender differences in the spillover effect from employee in-role to extra-role green behavior

**DOI:** 10.3389/fpsyg.2025.1516658

**Published:** 2025-04-28

**Authors:** Xinnan Wang, Jiafei Jin, Jialiang Xu, Mehedi Hasan Khan

**Affiliations:** School of Management, Harbin Institute of Technology, Harbin, China

**Keywords:** employee in-role green behavior, employee extra-role green behavior, reflective moral attentiveness, person-organization fit, gender difference

## Abstract

**Introduction:**

With the growing importance of green development, organizational research pointed that employee green behavior is an important micro foundation to addressing the environmental challenge. While past studies have categorized the in-role and extra-role green behavior as two dimensions of employee green behavior from the job performance perspective, they have overlooked the potential interaction between the two types of green behavior. This oversight may hamper organizations’ green efforts since deeper engagement in green behavior needs more psychological motivations compared to compliance with green management policies. According to cognitive consistency theory, this study explores employees’ psychology reactions to in-role green behaviors, and how these psychological changes induce extra-role green behaviors.

**Methods:**

Survey data collected in two times from 351 employees from 8 companies in China to assess the proposed hypothesis. SPSS 25 and Smart PLS 3.0 were used to test the theoretical model.

**Results:**

Results show that reflective moral attentiveness and person-organization fit mediate the spillover between in-role to extra-role green behavior. Moreover, these effects were moderated by employee gender: reflective moral attentiveness plays a more important role for women in the green behavior spillover process; conversely, in-role green behaviors lead to stronger person-organization fit for man than for woman.

**Discussion:**

This study provides unique insights into the potential interaction between the two types of green behavior. Furthermore, the psychological mechanisms of this behavioral change are different for male and female employees. Companies should take these differences into account when developing green management policies.

## 1 Introduction

Climate change significantly threatens companies’ long-term prosperity in almost every industry. Such growing public concern motivated governments worldwide to develop a series of regulation policies (e.g., carbon subsidies and tax) and environmental initiatives (e.g., the Paris Agreement) to encourage business efforts in greening (Castro-Casal et al., 2024; Hu et al., 2023). Greening the workplace and business practices have become a strategic goal for many companies. When green development is integrated into the social responsibilities and daily management processes, it is becoming increasingly recognized that employees’ behaviors are key to addressing the societal challenge of environmental sustainability (Aguinis et al., 2024; Zacher et al., 2023; Zhang S. et al., 2023).

On the one hand, companies introduced green duties in formal job description and required employees to complete work tasks in green ways, known as employee in-role green behavior (IRGB) (Zhang S. et al., 2023). For example, Alibaba, one of the world’s largest internet groups, launched a box recycling program in its logistics unit in 2019, asking its courier employees to recycle and reuse express delivery boxes after customers signed for them (Pconline, 2019). On the other hand, employee extra-role green behavior (ERGB) involves behaviors that may not be specified in formal job descriptions but help to make the organization and society more greener (Yang et al., 2025). ERGB often relies on employees’ voluntary participation, such as advocating for green practices or sharing green knowledge with colleagues (Palmié et al., 2023). Prior research suggested both IRGB and ERGB benefit organization’s green pursuits. However, recent studies suggest that it is difficult for employees to generate green behaviors that go beyond routine tasks (Yang et al., 2023). This difficulty may stem from a lack of attention to their intricacies and the potential green related behavioral spillover (Zhang S. et al., 2023).

A deeper understanding of the potential spillover and its underlying psychological mechanisms is essential for encouraging companies to promote green practices among employees. By revealing the change from passive to proactive green behavior, it can provide new insight into how employees make sense of in-role green behavior. This presents a key question to researchers and practitioners: how do green behaviors required as part of job responsibilities affect their psychology and motivate them to adopt more proactive green behaviors (Zacher et al., 2023)? Recent studies have highlighted the importance of exploring the role of positive subjective experiences by applying broaden theory (Meyers and Rutjens, 2022). Based on this view, we propose that the spillover of IRGB to ERGB requires affective and cognitive changes as an important foundation.

Additionally, although studies have explored the role of green climate (e.g., Dahiya, 2020), green human resource management (e.g., Yuan et al., 2023), etc. in enhancing IRGB from the organizational level, but most researches have focused on its antecedents; the consequences for employees remain under-explored. Investigating the consequences of IEGB is important and necessary. First, as employees are the primary agents in implementing green policies, taking an actor’s perspective on the effects of IEGB can provide valuable guidance for enhancing green performance and other desirable outcomes. Second, IRGB’s impact may extend beyond the environmental domain to influence broader employee work-related outcomes (Wang et al., 2023). Based on this, on the one hand, combined with “halo effect” and prosocial attributes of green behavior, we propose that IRGB has will induce positive moral attentiveness, making employees feel they are contributing to society in their actions. On the other hand, IRGB as a response to company’s green management policy, it will help employees to understand the policies and lead to higher value fit with company. Therefore, this study proposes the mediating effect of reflective moral attentiveness and person-organization fit to compensate for the current gap.

Finally, current study explores the trickle-down effect of leader green behaviors on subordinates at the team level (Shao et al., 2023), however, few have explored psychological responses within individuals and the differences between different groups of employees. Research indicates that work-related values and preferences are not always homogeneous across gender (Venkatesh et al., 2017). In green context, this difference may stem from the distinct ways in which men and women internalize organizational values and environmental norms (Shahzad et al., 2023). Kim et al. (2019) also pointed that gender is an important element influencing workplace green behavior. Thus, it is essential to investigate how gender influences the adoption and practice of green behavior. This can provide valuable insights for the design and development of green management policy.

In sum, our research offers three key contributions to the study of employee green behavior. First, we address the growing calls to explore the intricacies and the potential spillover effects of green-related behavior (e.g., Tang et al., 2023). By examining spillover across green behavior in the work domain, we provide new insight into the transition from passive to proactive green behaviors among employees. Second, we draw on cognitive consistency theory and conceptualize workplace green behavior as a process of aligning self-perception. In this sense, we advance employee green behavior research by identifying affective and cognitive psychological path in green behavior spillover effect. Third, by testing the moderating role of gender, our study provides a more comprehensive understanding of the behavioral spillover effect, contributing to a more nuanced understanding of the psychological and contextual factors influencing green behavior in the workplace.

## 2 Literature review and hypotheses development

### 2.1 Cognitive consistency theory and behavior spillover

The cognitive consistency theory (CCT) explains the importance of maintaining internal self-consistency between attitudes, beliefs, and behaviors (Abelson, 1968). According to this theory, people tend to act consistently across situations to maintain psychological balance and avoid uneasiness (Ahn and Kwon, 2020). In other words, people perceive themselves based on observing or imagining themselves behaving in a particular way. As a result, one’s actions serve as cues that help shape evaluative cognition, such as attitudes, norms, and values (Zhang S. et al., 2023). Over the years, research have shown that people are driven to achieve consistency between their attitudes, beliefs, and behaviors.

Research has also shown that the tendency to maintain consistency between past personal choices and future behavior has a profound impact in various settings (Zhang S. et al., 2023). Scholars have used cognitive consistency theory to study spillover in the personal domain. For instance, Lanzini and Thøgersen (2014) showed that individuals who have previously engaged in green purchasing are more inclined to participate in other green behaviors. Similarly, Palmié et al. (2023) found that employee with private pro-environmental orientation acts more pro-environmental behavior at work because people motivated by a desire to maintain consistency among their cognition and behavior. Thus, cognitive consistency theory provides a conceptual framework to explain why one’s behavior will lead to future behavioral change. We extend the application of cognitive consistency theory to the workplace domain and argue that employee in-role green behavior can be use as a source of knowledge about oneself in the green context, motivating one to keep to achieve further green goals.

### 2.2 The relationship between employee in-role and extra-role green behavior

Employee green behavior (EGB) is formally defined by Ones and Dilchert (2012) as “scalable actions and behaviors that employees engage in that are linked with and contribute to or detract from environmental sustainability.” Organizational psychologists have classified EGB into in-role (Bissing-Olson et al., 2013) and extra-role green behavior (Renwick et al., 2013). The essential distinction between these two categories of green behaviors is whether they are mandated, recognized, and rewarded by the organization (Ye et al., 2022). Since extra-role green behavior that goes beyond the formal job responsibilities depends on the employee’s self-determination (Daily et al., 2009), it requires stronger intrinsic motivation and costs extra resources (e.g., time and energy). Thus, in addition to changes in employee awareness and beliefs, a behavioral foundation is necessary.

According to cognitive consistency theory (Aronson and Carlsmith, 1962), acting consistently from one green behavior to another may be motivated by the desire to decrease cognitive dissonance and the discomfort it bears. A six-week tracking study conducted by Lanzini and Thøgersen (2014) pointed out that green purchasing behavior motivated by money produced a spillover effect to other pro-environmental behaviors. Zhang W. et al. (2023) pointed out that, due to the need to consolidate their self-cognition, completing work tasks in an eco-friendly way can prompt employees’ green advocacy. Thus, we argue that in-role green behavior is an important behavioral foundation for generating green behavior out of job responsibility. In order to maintain psychological consistency, IRGB can be transformed into ERGB.

*H1*: IRGB will positively influence ERGB.

### 2.3 The effect of reflective moral attentiveness in the spillover process

Reflective moral attentiveness (RMA) is one dimension of moral attentiveness, and it refers to the extent to which the individual regularly considers moral matters (Reynolds, 2008). From this view, issues are not objectively moral but rather constructed by individuals as moral (Reynolds, 2006). Due to the prosocial attribution of green behavior, RMA has been considered a factor strongly associated with employee green behavior (Zhang W. et al., 2023). From the stable and dispositional construct view, RMA is one of the important antecedents of extra-role green behavior (Afsar and Umrani, 2020). Moreover, in the literature on moral cognition development, it has been suggested that moral attentiveness can be developed through interventions and practice (Chung and Hsu, 2017). This developing perspective makes RMA an important factor in driving the transformation of employee green behaviors from in-role to extra-role.

First, to motivate employees’ green behaviors, companies start to provide employees with the necessary knowledge and abilities through incentives, penalties, and training programs (Pham et al., 2020). IRGB represents a response to formal organizational requirements, and it provides employees with cues that align with these proactive environmental policies for contributing to social responsibility. When employees perceive their work-related green actions as morally right or socially responsible, they may experience a sense of moral satisfaction. As Miao et al. (2020) suggested, RMA can be impacted by learning external information, hence, In-role green behavioral practices shaped stronger moral feelings among employees.

Second, RMA helps employees develop a concern for morality, leading to internalizing moral schema and values (Reynolds, 2008). When employees reflect on the moral dimensions of their actions, they are not only concerned with the external expectations set by the organization but also with their personal alignment to those values (Shahzad et al., 2023). According to cognitive consistency theory, individuals strive for congruence between their actions, beliefs, and values. This psychological drive for consistency leads employees to evaluate past behavior, such as engaging in green actions required by their role, and assess its alignment with their underlying moral values. In this sense, RMA provides employees with a moral framework that influences their subsequent behaviors. Such internal motivation enables IRGB to be continued. Thus, from a dynamic development perspective, we argue that RMA mediates the transition process from IRGB to ERGB.

*H2*: IRGB will positively influence RMA.

*H3*: RMA mediates the relationship between IRGB and ERGB.

### 2.4 The effect of person-organization fit in the spillover process

Person-organization fit (P-O fit) describes the compatibility between employees and their organization, and a shared value between individuals and the organizational environment or the ability of the two to meet the other’s needs (Chatman, 1989). In psychology, values can guide one’s attention, influence individual behaviors, and shape the interpretation of situational cues (Andersson et al., 2017). IRGB reflects the specific behaviors expected by organizations. Faced with these formal requirements from the organization, employees can choose to comply the required behavior or to resist (Silvia, 2005). Certainly, human nature is inherently and fundamentally driven to seek freedom and autonomy (Rosenberg and Siegel, 2018). When they engage in in-role green behavior, they will perceive that the organizational environment aligns with their personal values and fulfill their desire for autonomy that their jobs provide. Hence, we suggest that IRGB will enhance the alignment between employee and their organization.

Furthermore, ERGB is considered one type of organizational citizenship behavior, thus having a positive relationship with P-O fit (Venkatesh et al., 2017). Numerous studies on P-O fit support that employees whose values aligned with their organization’s values are more productive and actively participate in organizational citizenship behaviors (Erdogan et al., 2020). For example, Vilela et al. (2008) pointed out that a salesperson’s extra-role behavior is determined by his/her P-O fit. Green behaviors that go beyond task responsibilities are considered organizational citizenship behavior in the green context. Thus, P-O fit has also received attention in research on how to motivate employee’s green behaviors. Zhao et al. (2021) suggest that when employees’ personal needs align with the environmental cues provided by the organization, driven by intrinsic motivation and a sense of self-satisfaction, they are more likely to engage in ERGB.

Based on cognitive consistency theory, when employees follow organization-driven behaviors, norms and standards, the alignment between their behaviors and organizational values will strengthen their positive attitude toward their behavior (Raineri and Paillé, 2016). Thus, people’s perceptions of their past behavior influence their later behavioral decisions through cognitive changes. Since the organization does not formally require ERGB, it requires employees to generate value-level recognition. Thus, we argue that P-O fit mediates the transition process from IRGB to ERGB.

*H4*: IRGB will positively influence P-O fit.

*H5*: P-O fit mediates the relationship between IRGB and ERGB.

### 2.5 Gender difference in the spillover effect

In addition to exploring a complete comprehension of the mediating process between IRGB and ERGB, studies have emphasized the importance of identifying boundary conditions that shape the spillover processes (Shao et al., 2023). This is because employee affective (RMA) and cognitive (P-O fit) reactions brought on by IRGB are influenced by how they interpret and internalize the behavioral cues. Gender is an important demographic factor that strongly predicts individual moral and value differences (Shahzad et al., 2023). Thus, we suggest that there is a different effect for RMA and P-O fit in the spillover process of IRGB and ERGB for man and woman. This difference may stem from varying socialization processes and the distinct ways in which men and women internalize organizational values and environmental norms (De Cristofaro et al., 2021).

First, research has shown that women are often socialized to be more empathetic and socially responsible. This helps women better understand the moral practices inherent when engage in in-role green behavior (Shahzad et al., 2023), creating a stronger internal motivation to engage in extra behaviors. Moreover, In the theory of virtue ethics, it is suggested that women are more inclined to seek ‘internal goods’ that contribute to the well-being of others rather than focusing on ‘external goods’ related to personal possessions (MacIntyre, 1984; You et al., 2011). This internal-good-based rationality allows women to derive pleasure and satisfaction from behaviors that contribute to society’s well-being. Thus, we propose that women are more sensitive to the effects of reflective moral attentiveness between IRGB and ERGB than men.

Second, we also propose that the mediating impact of P-O fit is expected to be more pronounced in men than in women. Previous studies have shown that gender plays a role in how employees perceive the workplace, leading to different attitudes toward their organization (Yousaf et al., 2023). However, the potential moderate effect of gender on the relationship between P-O fit and its behavioral antecedents remains uncertain. Interaction psychology suggests that employees’ perceptions of P-O fit may not be determined solely by the attributes of the work environment. Instead, fit perceptions depend on both individual and work environment characteristics. Venkatesh et al. (2017) pointed out that women and men value different work outcomes, leading them to form different fit perceptions. For instance, when male employees engage in in-role green behaviors, they are more motivated to reflect on the organization’s new requirements and values. Since P-O fit evaluates how well an individual’s needs align with what the workplace provides, the perception changes of value fit brought on by IRGB are more critical for man than woman. Hence, we propose that:

*H6*: The positive influence of IRGB on RMA will be stronger for women than for men.

*H7*: The positive influence of IRGB on P-O fit will be lower for women than for men.

*H8*: The indirect influence of IRGB on ERGB through RMA will be stronger for women than for men; the indirect influence of IRGB on ERGB through P-O fit will be stronger for men than for women.

**Figure 1:**
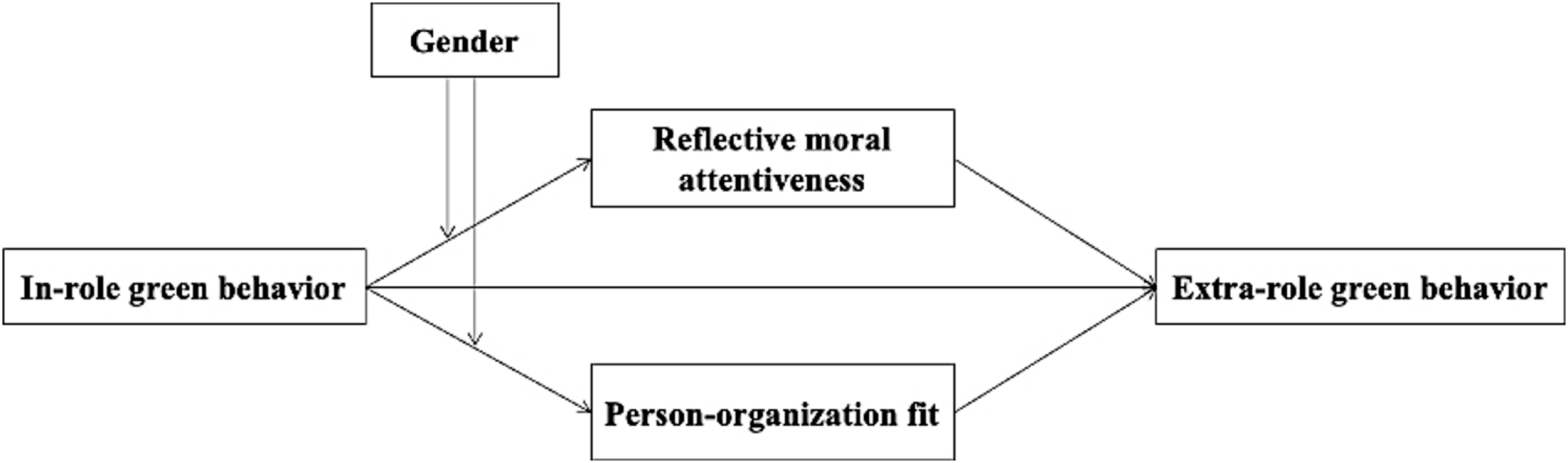
Research model.

The conceptual model (Figure 1) illustrates the relationships between research variables.

## 3 Methods

### 3.1 Sample and procedure

To test the hypotheses, we gathered data from front-line employees of eight companies in North China’s food processing, heating, and hospitality industries. This is because each of these companies has taken steps to green their business process under stricter environmental regulatory policies in recent years. And these three industries represent the three aspects of life services, travel, and food. In the heating industry, for example, especially in cities in northern China, the heating industry is a serious polluter of the environment and is strongly affected by environmental regulation. Thus, employee environmental performance is considered in the monthly performance appraisal system. Similarly, more and more hotels use environmentally friendly daily necessities and implement resource recycling. For the food processing industry, the green brand image will be shaped through the improvement of production processes. A common feature of green transformation in these industries is the need for front-line worker practices to maximize the effect of green policies.

We randomly recruited subjects at the scene of skill training programs at each company with the help of HR managers. After obtaining permission, we distributed the digital questionnaires to employees through HR managers. In order to guarantee voluntary engagement, participants were explicitly informed that the survey was entirely optional and they had the freedom to quit without facing any repercussions. Additionally, they were notified that the data would solely be utilized for academic research endeavors.

To minimize common method bias (CMB), we collected data in two waves. In April 2024, we conducted the first survey including control variable, the independent variable IRGB and one mediate variable P-O fit where 400 questionnaires were distributed. One month later, in May 2024, we conducted the second wave of data collection, the questionnaire includes the second mediator variable RMA and dependent variable ERGB. A total of 389 respondents returned the questionnaire. We used the first letter of given name and age to match the survey responses. After the second data collection, we excluded questionnaires that could not be matched and those with a short completion time (less than 1 min). We also excluded participants who failed the attention check (“Please choose ‘strongly disagree’ for this question.”). Finally, 351 valid responses were used for further analysis (87.8% response rate). Demographic characteristics of the respondents are shown in Table 1.

**Table 1:** The demographic characteristics (*n* = 351).

Respondents	Proportion	Respondents	Proportion
Gender		Education	
Man	45.3%	Bachelor degree	41.9%
Woman	53.7%	Master degree	14.8%
Age		PhD	3.7%
20–29 years old	27.1%	Others	39.6%
30–39 years old	30.8%	Tenure	
40–49 years old	23.1%	Less than one year	20.5%
50–59 years old	13.1%	1–5 years	35.9%
Over 60 years old	6%	6–10 years	13.4%
		11–20 years	16.5%
		More than 20 years	13.7%

### 3.2 Measures

Questions were rated on a five-point Likert scale (1 = strongly disagree, 5 = strongly agree) based on well-established measurement scales in previous research. To validate the accuracy of the scale item translation, an English version of the questionnaire was prepared, then translated into Mandarin and reintroduced into English. This method ensures the questionnaire maintains the original meaning (Saunders et al., 2009).

#### 3.2.1 In-role and extra-role green behavior

IRGB and ERGB were measured by Bissing-Olson et al. (2013). This scale included two dimensions of EGB (IRGB and ERGB); each dimension had 3 items. Participants were asked to reflect on the extent to which they were required to complete their work tasks in accordance with the company’s environmental requirements at time 1. An example item was “I performed tasks that are expected of me in environmentally-friendly ways.” This scale showed high reliability (*α* = 0.928). At time 2, participants were asked to self-evaluate their green behaviors beyond the job requirements. An example item was “I did more for the environment at work than I was expected to.” This scale showed acceptable reliability (α = 0.864).

#### 3.2.2 Reflective moral attentiveness

We assessed RMA via a 5 items scale developed by Reynolds (2008). This scale was adopted and validated by Zhang W. et al. (2023) in their study about EGB. An example item was “I regularly think about the ethical implications of my decisions.” This scale showed high reliability (α = 0.931).

#### 3.2.3 Person-organization fit

In the context of green behavior, cognitive fit is crucial because employees need to understand not only the organization’s green goals but also how their individual behaviors can support those goals. Thus, we choose the value dimension of P-O fit as measurement. A 3-item scale derived from Cable and DeRue (2002) was used to measure P-O fit. An example item was “My personal values match my organization’s values and culture.” This scale showed high reliability (α = 0.932).

#### 3.2.4 Controls

The control variables included gender, age, education and tenure, which are typically used in EGB research (Kim et al., 2017; Paillé and Francoeur, 2022; Ren et al., 2022).

### 3.3 Data analysis

SPSS 25 was used to enter and clean the data, and Smart PLS 3.0 was used to test the theoretical model. PLS-SEM has the advantage of maximizing the explained variance of endogenous variables (see Chin, 1998), it has been extensively used in theory testing and validation. Hence, we incorporated the SEM approach combined with PLS. PLS-SEM is frequently employed in many business fields (Venkatesh et al., 2017).

Following Hair et al. (2019), the structural model was analyzed. Since the data for this study was collected from a single source, the potential problem of common method bias (CMB) may exist (Podsakoff et al., 2012). Thus, we conducted tests for the variance inflation factor (VIF). The VIF values ranged from 1.071 to 1.297, indicating that collinearity issues are not a problem in this study (Diamantopoulos and Siguaw, 2006). Additionally, we conducted Harman’s single-factor test (Podsakoff et al., 2012). The total variance for a single factor was found to be 40.486%, less than 50%, suggesting that CMB did not significantly impact our data. Descriptive statistics and correlations for each variable are shown in Table 2.

**Table 2:** Descriptive statistics and correlation values.

Variables name	Mean	SD	1	2	3	4	5	6	7	8
1 Gender	1.55	0.50	1							
2 Age	2.4	1.19	−0.15**	1						
3 Education	2.41	1.37	−0.10	0.15**	1					
4 Tenure	2.67	1.34	−0.26**	0.55**	0.10	1				
5 IRGB	3.47	1.00	−0.06	0.10	0.04	0.10	1			
6 RMA	3.30	1.01	−0.15**	−0.04	−0.11*	−0.02	0.25**	1		
7 P-O fit	3.24	0.99	−0.06	0.03	0.01	0.07	0.45**	0.18**	1	
8 ERGB	2.96	1.12	−0.20**	0.06	0.03	0.15**	0.66**	0.34**	0.39**	1

**p < 0.01, *p < 0.05, IRGB, In-role green behavior; RMA, reflective moral attentiveness; P-O fit, person organization fit; ERGB, extra-role green behavior.

## 4 Results

### 4.1 Confirmatory factor analyses

Table 3 shows that the hypothesized four-factor model (CMIN/DF = 2.238, CFI = 0.978, TLI = 0.972, RMSEA = 0.059, SRMR = 0.0385) performed better than the other models in confirmatory factor analyses (CFAs).

**Table 3:** Confirmatory factor analysis.

Models	CMIN/DF	GFI	TLI	CFI	SRMR	RMSEA	Alternative models
One-factor model	31.428	0.451	0.321	0.426	0.2182	0.295	IRGB+RMA + P-O fit+ERGB
Two-factor model	21.262	0.557	0.548	0.623	0.2540	0.241	IRGB+RMA, P-O fit+ERGB
Two-factor model	18.288	0.649	0.614	0.678	0.1957	0.222	IRGB+ERGB, RMA + P-O fit
Three-factor model	5.270	0.843	0.905	0.923	0.0688	0.110	IRGB+ERGB, RMA, P-O fit
Four-factor model	2.238	0.939	0.972	0.978	0.0385	0.059	IRGB, RMA, P-O fit, ERGB

IRGB, in-role green behavior; RMA, reflective moral attentiveness; P-O fit, person organization fit; ERGB, extra-role green behavior.

### 4.2 Assessment of measurement model

#### 4.2.1 Assessing convergent validity

**Figure 2:**
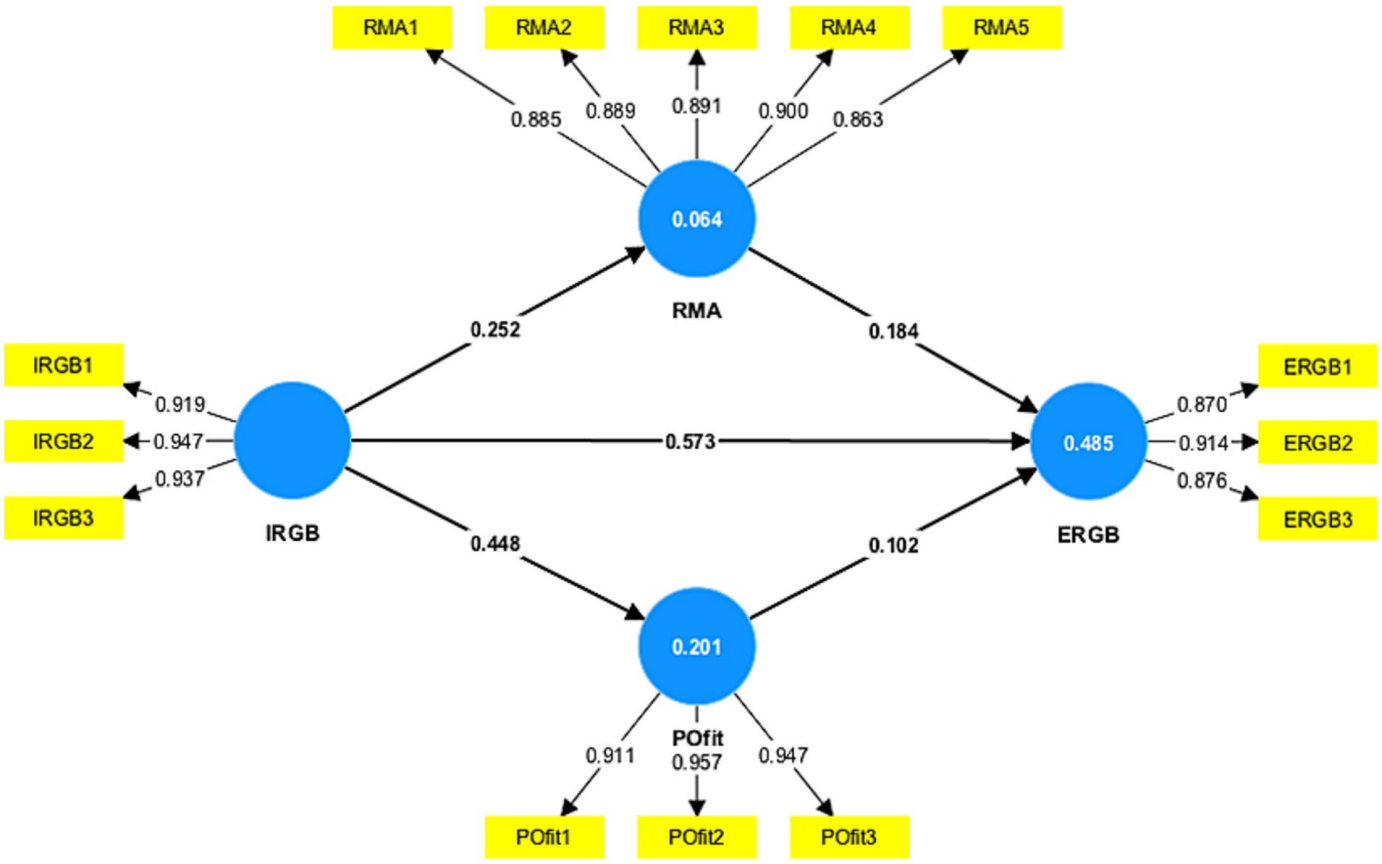
Path estimates.

For reliability and convergent validity, we examined item factor loadings (Figure 2), Cronbach’s alpha, composite reliability (CR) and average variance extracted (AVE) (Hair et al., 2017). Table 4 shows that all factor loadings were higher than the recommended threshold of 0.8. As shown in Table 4, values of AVE are above 0.5, and values of CR are above 0.7. Therefore, the convergent validity of the constructs is satisfactory (Fornell and Larcker, 1981).

**Table 4:** Measurement constructs and convergent validity.

Measurement constructs and indicators	Loadings	α	AVE	CR
IRGB		0.928	0.874	0.928
In my work, I adequately completed assigned duties in environmentally-friendly ways.	0.919			
In my work, I fulfilled responsibilities specified in my job description in environmentally-friendly ways.	0.947			
In my work, I performed tasks that are expected of me in environmentally-friendly ways.	0.937			
RMA		0.931	0.784	0.931
I regularly think about the ethical implications of my decisions.	0.885			
I think about the morality of my actions almost every day.	0.889			
I often find myself pondering about ethical issues.	0.891			
I often reflect on the moral aspects of my decisions.	0.900			
I like to think about ethics.	0.863			
P-O fit		0.932	0.881	0.932
The things that I value in life are very similar to the things that my organization values.	0.911			
My personal values match my organization’s values and culture.	0.957			
My organization’s values and culture provide a good fit with the things that I value in life.	0.947			
ERGB		0.864	0.787	0.864
I took a chance to get actively involved in environmental protection at work.	0.870			
I took initiative to act in environmentally-friendly ways at work.	0.914			
I did more for the environment at work than I was expected to.	0.876			

#### 4.2.2 Discriminant validity

Fornell–Larcker criterion and Heterotrait Monotrait Ratio (Henseler et al., 2015) were tested to confirm that each construct was statistically different from the others (Hair et al., 2019).

##### 4.2.2.1 Fornell–Larcker criterion

According to Table 5, each diagonal value (square root of AVE) is higher than the variance shared with the other constructs (Henseler et al., 2015). This indicates the validity and internal consistency of the measures utilized in this study.

**Table 5:** Discriminant validity test.

Construct	Fornell–Larcker criterion				Heterotrait Monotrait Ratio (HTMT)			
	IRGB	RMA	P-O fit	ERGB	IRGB	RMA	P-O fit	ERGB
IRGB	0.935							
RMA	0.252	0.886			0.265			
P-O fit	0.448	0.181	0.939		0.482	0.193		
ERGB	0.665	0.347	0.392	0.887	0.739	0.384	0.437	

The value in the italic face are the square root of AVE. IRGB, in-role green behavior; RMA, reflective moral attentiveness; P-O fit, person organization fit; ERGB, extra-role green behavior.

##### 4.2.2.2 Heterotrait Monotrait Ratio (HTMT)

According to Table 5, the values are between 0.193 and 0.739, all below 0.85, confirming the discriminant validity of the constructs in this study.

### 4.3 Assessment of structural model

#### 4.3.1 R square

The cut-off values for *R*^2^, according to Chin (1998), are 0.19, 0.33, and 0.67, indicating weak, moderately strong, and substantially strong, respectively. The results showed that IRGB, RMA and P-O fit collectively explained 48.1% of the variance in ERGB, falling within the moderately strong range.

#### 4.3.2 Q square

We computed *Q*^2^ by blindfolding to evaluate the structural model. *Q*^2^ establishes the predictive relevance of the endogenous constructs. A *Q*^2^ above zero shows the model has predictive relevance (Hair et al., 2017). In this study, Q^2^ values of RMA (0.046), P-O fit (0.174) and ERGB (0.373) are all above 0, fulfilling statistical requirements.

#### 4.3.3 Hypotheses testing

We validated the hypotheses through the bootstrapping procedure, following the recommendations of Preacher and Hayes (2004, 2008). Table 6 shows the results of bootstrapping.

**Table 6:** Results of the structural model.

Hypothesis	Coefficient	SE	*T*-values	*p*-values	95% Confidence interval		Support
					Lower level	Upper level	
Results of direct effects							
H1: IRGB - > ERGB	0.573	0.046	12.486	0.000	0.478	0.660	Yes
H2: IRGB - > RMA	0.252	0.055	4.578	0.000	0.148	0.361	Yes
H3: RMA - > ERGB	0.184	0.045	4.046	0.000	0.096	0.275	Yes
H4: IRGB - > P-O fit	0.448	0.047	9.548	0.000	0.352	0.535	Yes
H5: P-O fit - > ERGB	0.102	0.046	2.200	0.028	0.010	0.195	Yes
Results of indirect effects							
H3: IRGB - > RMA - > ERGB	0.046	0.015	3.005	0.003	0.021	0.080	Yes
H5: IRGB - > P-O fit - > ERGB	0.046	0.022	2.122	0.034	0.005	0.090	Yes
Results of total indirect effects							
IRGB - > ERGB	0.092	0.025	3.739	0.000	0.047	0.144	Yes

IRGB, in-role green behavior; RMA, reflective moral attentiveness; P-O fit, person organization fit; ERGB, extra-role green behavior.

##### 4.3.3.1 Analysis of direct effects

IRGB significantly positively impacts ERGB (*β* = 0.573, CI = 0.478, 0.660), supporting H1. Then, IRGB significantly increased employee reflective moral attentiveness (*β* = 0.388, CI = 0.281, 0.493), providing support for H2. Reflective moral attentiveness significantly increased ERGB (*β* = 0.184, CI = 0.096, 0.275), which initial support for the first mediation path. For H3, IRGB significantly increased P-O fit (*β* = 0.448, CI = 0.352, 0.535) and P-O fit had a positive impact on ERGB (*β* = 0.102, CI = 0.010, 0.195), which provided initial support for the second mediation relationship.

##### 4.3.3.2 Analysis of indirect effects

As shown in Table 6, RMA mediates the relationship between IRGB and ERGB (indirect effect = 0.046, SE = 0.015, 95% CI = 0.021, 0.080), thus H4 was confirmed. Moreover, P-O fit also mediates the relationship between IRGB and ERGB (indirect effect = 0.046, SE = 0.022, 95% CI = 0.005, 0.090). Since the confidence interval does not contain zero, H5 was supported.

##### 4.3.3.3 Analysis of moderator

We analyzed the influence of gender by conducting a multigroup analysis as outlined by Henseler et al. (2009). Initially, the structural model was tested separately for men and women, and the path coefficients are then compared across these two groups. The following formula (Sia et al., 2009) was used to calculate the *t*-value and to evaluate the significance levels of these differences. Previous research has shown that multigroup PLS is an effective method for examining subgroup differences (e.g., Keil et al., 2000). Table 7 shows the results from the subgroup analysis based on gender.


$$S_{\text{pooled}}=\sqrt{\frac{{\left(N_{1}-1\right)}^{2}}{N_{1}+N_{2}-2}\times SE_{1}^{2}+\frac{{\left(N_{2}-1\right)}^{2}}{N_{1}+N_{2}-2}\times SE_{2}^{2}}$$

$$t_{\text{spooled}}=\frac{PC_{1}-PC_{2}}{S_{\text{pooled}}\times \sqrt{\frac{1}{N_{1}}+\frac{1}{N_{2}}}}$$

**Table 7:** Path comparison statistics between woman and man.

Path	Woman (*n* = 192)		Man (*n* = 159)		*T*_spooled_	Support
	Path coefficient	*t*-value	Path coefficient	*t*-value		
H6: IRGB - > RMA	0.360	5.303***	0.115	1.262^NS^	2.203*	Yes
H7: IRGB - > P-O fit	0.360	5.243***	0.542	8.673***	−1.920^NS^	No
H8: IRGB - > RMA - > ERGB	0.107	3.771***	0.013	0.970^NS^	2.859*	Yes
H8: IRGB - > P-O fit - > ERGB	−0.001	0.049^NS^	0.080	2.114*	−1.896^NS^	No

***p < 0.001, **p < 0.01, *p < 0.05, NS, statistically not significant; IRGB, in-role green behavior; RMA, reflective moral attentiveness; P-O fit, person organization fit; ERGB, extra-role green behavior.

Where,

*S_pooled_* = pooled estimator for the variance.

*t* = t-statistic with N_1_ + N_2_-2.

*SE* = standard error of path in structural model of gender i.

*PC_i_* = path coefficient in structural model of gender i.

**Figure 3:**
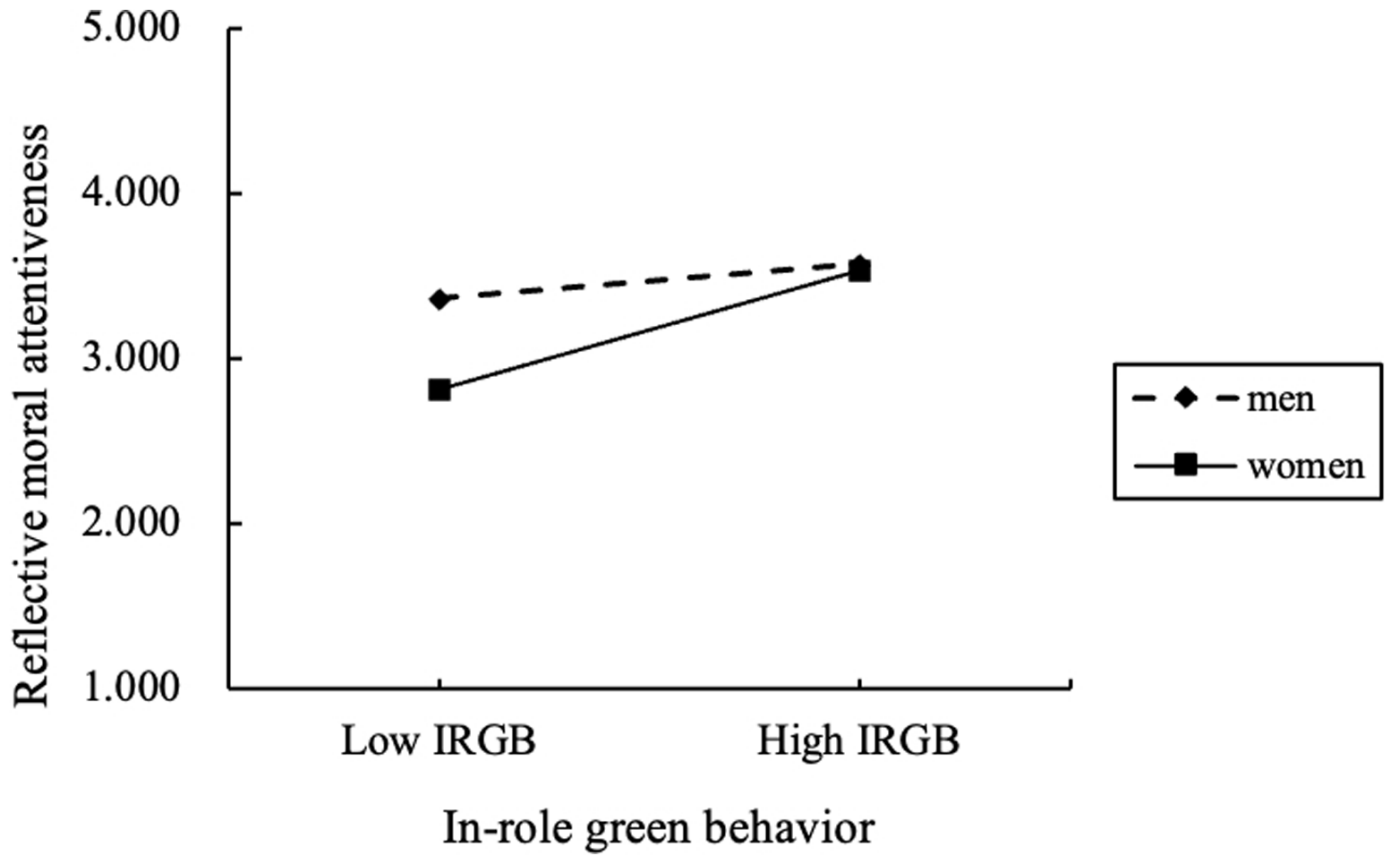
IRGB—gender interaction for RMA.

First, the multigroup analysis revealed that in the woman group, IRGB has a significantly positive impact on RMA (path coeff. = 0.360, *t*-value = 5.303***), while in the man group, there is no significant positive effect (path coeff. = 0.115, *t*-value = 1.262), and the path coefficients between the two groups have significant difference (*T*_spooled_ = 2.203*); thus H6 was supported. The moderating relationship was plotted significantly in Figure 3, revealing that the relationship between IRGB and RMA was more positive in women than in men. Simple slope tests also confirmed the results that the relationship was stronger in women group.

**Figure 4:**
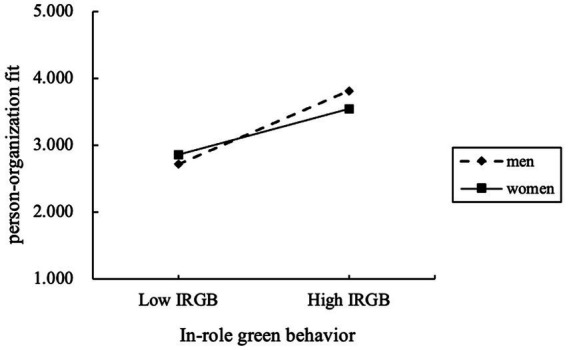
IRGB—gender interaction for P-O fit.

Second, for H7, the positive effect of IRGB on P-O fit is significant in both the woman (path coeff. = 0.360, *t*-value = 5.243***) and man (path coeff. = 0.542, *t*-value = 8.673***) groups. The moderating relationship was plotted significantly in Figure 4. Although the path coefficient in the man group is higher than in the woman group, there is no significant difference between the two groups (*T*_spooled_ = −1.920); hence H7 is not supported.

Third, for H8, the path coefficient from IRGB to ERGB via RMA in the woman group was significantly stronger than that in the man group (*T*_spooled_ = 2.859*, woman path coeff. = 0.107, man path coeff. = 0.013). However, the mediation effect of P-O fit was significant in the man sample and wasn’t significant in the woman sample; the difference in this indirect effect between man and woman wasn’t significant (*T*_spooled_ = −1.896, woman path coeff. = − 0.001, man path coeff. = 0.08); therefore, our results partially supported H8.

## 5 Discussion

### 5.1 Direct and indirect path analysis

Although such behavioral spillover has been studied at least since the 1970s (Lanzini and Thøgersen, 2014), barely any study discussed what kind of spillover effect IRGB will produce in the employee’s work context. This study provides insight into the potential transition process between two types of green behavior. According to the results, H1 (IRGB - > ERGB) is accepted as IRGB positively predicts ERGB. This result is consistent with a previous study by Zhang S. et al. (2023). They examined how required employee green behavior is connected to a type of ERGB, namely green advocacy. Our result indicates that the spillover effect of green behaviors also exists in the work context, providing new empirical support for the study of green behavior outcomes. Employees can act consistently across various green behaviors, leading to positive spillover from one behavior to another.

H2 (IRGB - > RMA) reveals that IRGB can improve employees’ attention to the moral content in individual behaviors and decisions, leading an individual towards the reflexive moral course of action. This result is consistent with the view of the development of morals, which suggests that in response to the organization’s external intervention, green behaviors generated by employees in accordance with their new responsibilities provide a behavioral basis for the formation of RMA. Moreover, H3 (IRGB - > RMA - > ERGB) indicates that increased RMA promotes the transition from IRGB to ERGB. This finding is important because few studies have focused on the role of RMA between IRGB and ERGB. Although previous research has discussed RMA’s mediating effect in the relationship between organization’s green management policies and green behaviors, it mainly focused on the organizational level. Our study provides a possible explanation from an individual behavioral level, suggesting that RMA allows employees to extend their green behaviors beyond their responsibilities.

H4 (IRGB - > P-O fit) reveals that IRGB also affects employees’ perceptions of how values fit between themselves and the organization. Previous research has illustrated the contribution of P-O fit to employee workplace behaviors or voluntary green behaviors, but our results suggest that IRGB may be an antecedent in forming P-O fit. This indicates that organizations can attract new employees with similar green ideologies and alter existing employees’ values through intervention policy. When practicing IRGB, it brings direct cognitive judgments for employees. Moreover, the fifth hypothesis (IRGB - > P-O fit - > ERGB) shows that employees with increased value fit will perform more ERGB. We first discussed the role of P-O fit in two green behavior transitions, explaining the mediating mechanism from a cognitive reaction perspective.

### 5.2 Moderate path analysis

The sixth to eighth hypotheses reveal the moderate effect of gender in the relationship between IRGB and its outcomes. This finding aligns with the perspective on gender differences in morality but has not been examined in the green context before. Thus, these are the most valuable and interesting findings of this study. First, there is a significant difference in the reactions to IRGB between man and woman: the direct influence of IRGB on RMA is much stronger in woman than in man (H6), however, IRGB brings increased P-O fit for both woman and man. This indicates that the moral perception of IRGB is more critical for woman, and this will influence women’ decisions of subsequent behavior. Consistent with prior studies (Shahzad et al., 2023), it has been found that man and woman have distinct moral strategies.

Second, we also found the moderated mediating effect. Compared to woman, the mediating effect of IRGB on ERGB through RMA was insignificant in men. This indicates that moral perceptions only influence the behavioral transition for women. It provides insights for organizations to make targeted changes in employees’ green behavior. For the mediating effect of P-O fit, there is no gender difference in this mediating path; thus, our results partly support hypothesis 8. This may be because various vital factors influence the shaping of person-organization fit, and compared to job values and criteria, environmental vision is not the most central factor. These findings shed light on gender roles in designing and changing employees’ green behaviors.

## 6 Conclusion

This study operates under the hypothesis that employee in-role green behavior can transform into extra-role green behavior through affective and cognitive paths, and the mediate effect is different for men and women. According to the findings of our study, reflective moral attentiveness and person-organization fit mediate the spillover process between two types of green behaviors. We provide evidence that IRGB and ERGB may not be generated simultaneously since ERGB needs stronger motivation. In addition, one of our goals is to determine whether the factors that encourage ERGB differ for man and woman employees. Our results indicate that the mediation effect of moral affective in the spillover process is more substantial for woman.

### 6.1 Theoretical implications

The study has some theoretical contributions to the extant body of knowledge. First, we explore the intricacies between two types of green behavior, which aligns with the recent recommendations in the HRM literature (Norton et al., 2015; Zhang S. et al., 2023). This study offers novel insights that inform whether there is a positive spillover effect between IRGB and ERGB. In the green context, this continuous and spillover effect between green behaviors has been demonstrated a lot in consumer behavior studies (Cornelissen et al., 2008). However, it has rarely been validated in the context of employee behavior. Although previous studies have clearly distinguished IRGB and ERGB and analyzed them as separate outcome variables, we provide a new insight that ERGB requires stronger psychological motivation and a certain behavioral foundation. Furthermore, due to increasing public and governmental regulatory pressures (Nyilasy et al., 2014), green development strategy has emerged as a crucial aspect of ethical business practices. By theorizing and testing the effect of IRGB in inspiring broader forms of green behavior, this study provides a new insight into the vital role of in-role green behavior foundation in business ethics research.

Second, this study applies cognitive consistency theory (Abelson, 1968) to examine the role of affective and cognitive reactions in the spillover mechanisms between green-related behaviors, providing a more nuanced explanation of green behavioral outcomes at the individual level. Our findings provide evidence that people tend to act consistently across their past and subsequent green behaviors (Thomas et al., 2016). Empirical studies conducted in the marketing domain have shown that people tend to engage in recycling after being encouraged to green purchasing (Lanzini and Thøgersen, 2014). However, most studies focus on the personal domain. We extend cognitive consistency theory to explain the spillover between workplace green behaviors by proposing in-role green behavior as a foundation of psychological reinforcement processes. When employees engage in these green behaviors, they start to see themselves as individuals who are environmentally responsible, which creates a psychological alignment between their actions and their self-concept. This alignment serves as a cognitive cue that helps shape their broader environmental attitudes, and demonstrate the utility of the theory in organizational environmental management.

Third, our study fills the gaps in the spillover boundary condition between green behaviors. By verifying the moderating role of gender, we extend the understanding of the differences in the psychological outcomes brought by in-role green behaviors for man and woman employees. Shahzad et al. (2023) pointed out that gender is considered a key demographic factor that strongly predicts individual values and moral differences. Moreover, we provides a supplement to Zhang S. et al.’s (2023) study. They conducted a study on the spillover effects of green behaviors with a sample of the Chinese manufacturing industry, but due to the characteristics of the industry, 97% of the subjects were men. Therefore, they also point that the need to study gender differences in the future. Our findings suggest that, performing work duties in a greener way elicits more morally psychological feelings for female employees, encouraging them to be more inclined to participate in ERGB. For male employees, in-role green behaviors prompt increased reconsideration of how well their values fit with the organization, generating a positive spillover effect from this path. This moderator effect might explain prior studies’ inconsistent spillover (positive/negative) of green behaviors (Tiefenbeck et al., 2013). These findings respond to the call by Francoeur et al. (2021) to have a deep understanding of the outcomes of workplace green behavior and to provide more specific insights to motivate positive spillover.

### 6.2 Practical implications

Our study has several practical implications. First, organizations can “green” their employees by green training programs and gradually adding green requirements to current work tasks. It is important for organizations to take into account the ethical consequences of employee-level attributes and pay attention to the consistency between employees’ perceived values related to morality and organization’s vision (Zhang et al., 2022). For example, HR managers can launch morality-driven sustainability workshops that highlight the ethical implications of green practices. Encourage employees to reflect on the moral significance of their work in relation to broader environmental impacts, fostering a stronger sense of responsibility and engagement.

Second, strengthen person-organization fit through green organizational culture. For example, develop a green mission statement or set of core values. A well-defined mission statement not only outlines the company’s environmental objectives but also signals to employees that sustainability is integral to the organization’s long-term vision and operational identity. When employees feel that their personal values align with the organization’s green objectives, the psychological feeling of consistency brought by IRGB is more likely to drive deeper engagement in green behaviors. A strong sense of person-organization fit fosters intrinsic motivation to act in ways that support the organization’s environmental goals.

Third, address gender differences through tailored communication. On the one hand, frame environmental initiatives in ways that appeal to the intrinsic motivations of women, such as emphasizing collective social responsibility or the moral impact of green behaviors. On the other hand, for men, messages could focus on the practical benefits of green behaviors, such as efficiency gains or organizational success linked to sustainability. This includes highlighting how sustainable practices contribute to organizational success, improve efficiency, or enhance job performance. Men may be more responsive to messages that link green behaviors to tangible outcomes such as cost savings, productivity gains, or competitive advantage. Tailored communication strategies ensure that both women and men feel valued and understood, thereby fostering greater overall engagement in sustainability efforts across the organization.

### 6.3 Limitations and future research directions

This study also has certain limitations. First, we conducted a self-report survey; thus, common method bias (CMB) is a potential concern. Although we collected independent, mediating and dependent variables in 2 times to minimize the impact of CMB, future studies should use more objective data (such as observations or archival data).

Second, we examined the model only once and did not track how employees’ psychological feelings changed over time. Therefore, we recommend that future studies try to use longitudinal methods to explore the long-term effects of gender differences in green behavior and their evolution over time (Zacher et al., 2023). Additionally, suggest using experimental designs to test for causality and determine the underlying mechanisms of the observed spillover effects. This approach would strengthen the understanding of the relationship between in-role and extra-role green behaviors.

Third, the empirical data collected in different industries in China may not be fully representative of the broader population. Therefore, future research should aim to strengthen the robustness of variable relationships across a wider scope of settings using alternative analytic measures and methods. Furthermore, gender norms and workplace expectations may influence behavioral patterns differently across cultures, and thus the moderating role of gender may perform differently in Western versus Eastern cultural contexts. To rule out limitations due to the sample, socio-cultural factors (such as Taoism in traditional Chinese culture) should be discuss with a larger-scale study.

Fourth, although this study discussed the mediating mechanisms of IRGB to ERGB transformation and chose gender as an individual-level boundary condition, other potential individual and contextual moderators should also be explored in the future. Since ERGB require employees to invest additional time and resources, not all employees may feel compelled to act consistently. The stress of job duties combined with green behaviors may lead to more cognitive stress, leaving the role of personal traits, autonomy, etc., to be discussed. For instance, research could explore how employees’ proactive personality, sense of autonomy, perceived workload, or organizational support influences their willingness to continuously engage in extra-role green behaviors.

## Data Availability

The raw data supporting the conclusions of this article will be made available by the authors, without undue reservation.
